# Rectal stenosis after circular mechanical anastomosis; the influence of stapler size

**DOI:** 10.1007/s00464-024-11306-8

**Published:** 2024-10-14

**Authors:** Núria Llorach-Perucho, Ladislao Cayetano-Paniagua, Pau Esteve-Monja, Albert Garcia-Nalda, Josep Bargalló, Xavier Serra-Aracil

**Affiliations:** 1 Coloproctology Unit, General and Digestive Surgery Service, Parc Tauli University Hospital, Sabadell. Institut d’investigació i innovació Parc Tauli I3PT-CERCA, Sabadell (Barcelona), Spain; 2 Department of Surgery, Universitat Autònoma de Barcelona, 08208 Sabadell (Barcelona), Spain; 3 Coloproctology Unit, Consorci Sanitari de Terrassa, Terrassa (Barcelona), Spain; 4 Institut d’investigació I Innovació Parc Tauli I3PT-CERCA, Sabadell (Barcelona), Spain

**Keywords:** Benign anastomotic stenosis, Colorectal anastomosis, Circular mechanical staplers, Low anterior resection of the rectum

## Abstract

**Background:**

The incidence of benign anastomotic stenosis (BAS) after radical surgery for rectal cancer ranges from 2 to 30%. There are few data regarding the factors related to its occurrence. One of these factors is the diameter of the circular mechanical staplers (CMS) used.

**Methods:**

Observational study with prospective data recording of consecutive patients with non-disseminated rectal cancer operated on at two hospitals with special dedication to rectal cancer. Patients underwent low anterior resection (LAR) of the rectum with colorectal anastomosis created using CMS of diameters of either 28–29 or 31–33 mm. The primary endpoint was BAS. Secondary variables were demographic and patient-dependent data, and preoperative, intraoperative, immediate postoperative and mid-term data. The incidence of BAS was compared in the groups in which the different stapler diameters were used.

**Results:**

Between 2012 and 2022, 239 patients were included. BAS was recorded in 39 (16.3%). In the analysis of factors related to its occurrence, the only significant variable was stapler diameter (*p* = 0.002, 95% CI 7.27–23.53), since rates of BAS were lower in the 31–33 mm group. Similarly, in the logistic regression analysis, stapler size was not associated with postoperative complications or anastomotic dehiscence (OR 3.5, 95% CI 1.2–10.5). Comparing stapler groups, BAS was detected in 35 of 165 patients (21%) in the 28–29 mm group but in only four out of 74 (5.6%) in the 31–33 mm group (*p* = 0.002, 95% CI 7.27–23.53). Ileostomy closure took longer and was less frequent in the 28–29 mm group.

**Conclusions:**

The rate of BAS after LAR was not negligible, since it was recorded in 39 of 239 patients (16.3%). The use of a 31–33 mm CMS was associated with a lower incidence of BAS. Therefore, the use of larger staplers is tentatively recommended; however, clinical trials are now required to confirm these results.

**Graphical abstract:**

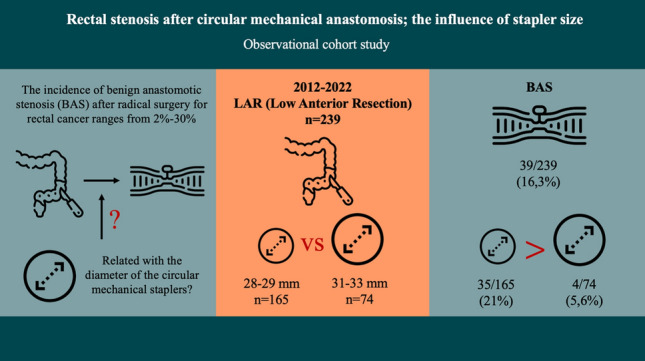

Colorectal anastomoses after resection of the left colon or rectum may present complications, such as dehiscence and bleeding early in the postoperative period and anastomotic stenosis at later stages [1]. Suture dehiscence is the most feared postoperative complication of colorectal surgery with anastomosis. Its incidence varies widely from study to study, between 1 and 28% [2]; the more distal the location, the more likely it is to occur. Its clinical representation ranges from severe forms of septic shock to more latent forms such as paralytic ileus or abdominal pain. Risk factors include preoperative malnutrition, comorbidity, smoking, immunosuppression or severe intraoperative blood loss requiring transfusion [3].

Bleeding from the anastomosis is another acute complication. Although alarming, it is usually of less clinical relevance, since most incidents are self-limiting and do not entail any clinical repercussions for the patient. However, some cases of bleeding are massive and may be life-threatening. The incidence of severe bleeding ranges from 0.5 to 4.2% and can make diagnosis and treatment difficult [4].

One of the medium-term complications of colorectal anastomoses is benign anastomotic stenosis (BAS), reported in between 2 and 30% of cases. This percentage varies greatly due to the scarcity of studies in the literature and a lack of consensus on the definition of this complication [5, 6]. BAS is associated with ongoing morbidity, the need for additional diagnostic and therapeutic procedures, and hospital admissions [1]. In the medium term, it translates into poor functional results, heightens the risk of permanent colostomy, and increases the likelihood of cancer recurrence [7–9].

Colorectal stenosis is the most common late complication after a rectal surgery, so this type of surgery is considered a risk factor for its occurrence. Other risk factors described are male sex, neoadjuvant treatment, preoperative obesity, sepsis, irradiation, incomplete donuts, and in the postoperative period, anastomotic leak, pelvic infection, and radiotherapy. The surgical technique is a well-recognized factor that influences the outcome of an anastomosis, including the type of anastomosis and its diameter. Insufficient colonic preparation, inadequate blood supply to the colonic and rectal stump, and tension at the site of the anastomosis caused by absence of mobilization of the splenic flexure all increase the incidence of anastomotic leakage, pelvic sepsis, and subsequent stenosis [9–11].

Regarding the diameter of the anastomosis, few studies have evaluated BAS as a function of the diameter of the circular mechanical stapler (CMS) used. A recent study by Reif de Paula et al. [12] assessed the influence of the diameter of the anastomosis in left colon and rectal resections, finding that the use of 25–29 mm staplers was associated with a higher rate of anastomotic stenosis than the use of 30–33 mm staplers.

In view of the wide variability of the rate of BAS, the lack of a widely agreed definition and the relationship with triggering factors, especially its relationship with the diameter of the CMS, the present study was designed to test the hypothesis that the frequency of BAS after the creation of anastomoses with CMS in the middle and lower rectum is non-negligible and that it is related to the diameter of the CMS used.

The primary aim of the study was to determine the rate of BAS after the use of a CMS in radical rectal cancer surgery and its relationship with the stapler diameter. The secondary aim was to identify any other factors related to BAS.

## Materials and methods

### Study design

Observational study with prospective recording of data from consecutive patients with rectal cancer at two hospitals who underwent low anterior resection (LAR) of the rectum with colorectal anastomosis using CMS measuring 28–29 mm or 31–33 mm in diameter (the choice of stapler used was based on the subjectivity and experience of the surgeon).

The present study was approved by both local institutional ethics committees (CEIC: 2024/5002 and 02-24-102-019), and complied with the criteria of the Declaration of Helsinki. The STROBE guidelines for observational studies were followed [13].

### Patients. Patient selection

All patients were operated on by surgeons from the Coloproctology Units of the Universitari Parc Taulí Hospital in Sabadell (5) and the Consorci Sanitari in Terrassa (4) between June 2012 and June 2022. The selected patients followed the protocols of the international guidelines for rectal cancer [14, 15] and their cases were discussed by the multidisciplinary colorectal cancer committees of both hospitals. The follow-up of the patients was based on the NCCN rectal cancer guidelines, which includes endoscopic evaluation, allowing the diagnosis of stenosis that do not present clinical symptoms for the patient [14].

*Inclusion criteria*. Adult patients (over 18 years of age) who underwent scheduled surgery for non-disseminated middle or lower rectal cancer (lower margin less than 10 cm from the anal verge) by total mesorectal excision (TME) with or without protective ileostomy at the discretion of the surgeon; circular mechanical anastomosis; open or minimally invasive approach (laparoscopy or robot-assisted); follow-up of at least 1 year after rectal surgery.

*Exclusion criteria*. Pregnancy; patients with disseminated disease (M1); palliative surgery; emergency surgery; absence of colorectal anastomosis (Hartmann procedure or abdomino-perineal resection); manual anastomosis, or mechanical anastomosis other than CMS.

### Patient preparation and surgical technique

All patients underwent mechanical colon preparation. According to the protocol of each center, antibiotic prophylaxis was administered during anesthetic induction and thromboembolic prophylaxis.

Techniques performed: LAR: open, laparoscopic LAR was performed applying the classic standards described by the COLOR II study [16] and Heald [17]. TaTME: performed using transanal TEO TME, as described by Serra-Aracil [18].

### Primary endpoint

Presence of BAS with a minimum follow-up of 1 year and data on the diameter of the CMS used.

BAS was divided into three groups according to the Truong classification [19]: grade I, anastomosis diameter between 10 and 20 mm and/or the presence of occasional abdominal symptoms; grade II, anastomosis diameter between 5 and 9 mm and/or in the presence of frequent abdominal symptoms; grade III, anastomosis diameter less than 5 mm and/or the presence of abdominal symptoms of intestinal occlusion.

### Secondary endpoints

Patient-dependent and demographic variables: sex, age, comorbidities [smoking, diabetes, vasculopathy, BMI (body mass index), ASA, preoperative albumin, chronic use of corticosteroids or immunosuppressants], surgical indication and neoadjuvant therapy.

Surgical variables: date of intervention, technique performed, approach, anastomosis diameter, stapler used, type and location of the anastomosis, surgical time and evidence of intraoperative anastomotic leak.

Immediate postoperative variables (30 days): postoperative complications defined according to the Clavien-Dindo classification [20], clinically relevant when greater than II (IIIa, IIIb, IVa, IVb and V) and the Comprehensive Complication Index (CCI) [21]. Postoperative anastomotic leak was classified according to the definition of the International Study Group of Rectal Cancer [22] in three groups: A, not requiring active treatment; B, requiring active non-surgical treatment; C requiring active surgical treatment.

Postoperative variables at medium term (from 30 days to at least 1 year): presence of ileostomy, ileostomy closure and date (if applicable), presence of BAS, BAS grade according to Truong classification [19] and date of last follow-up.

### Sample size

To establish the power of the sample, the sample size was calculated for a proportion in an infinite population. To do this, the formula *n* = (*Z*_1−*α*_)^2^*p*q/*d*^2^was applied, where 1 − *α* is the confidence level (0.95), *Z*_1 − α_ is 1.96, *p* the approximate proportion of the phenomenon under study in the reference population, *q* = proportion of the reference population that does not present the phenomenon under study (1 − *p*). *d* = level of precision (0.05). For an estimated proportion of stenosis in the population of 15% [23] and a loss of 10% the sample size calculated is 210 patients [24].

### Statistical analysis

SPSS version 26 (SPSS, Inc., Chicago, IL) was used. Due to the prospective nature of data collection, there were few missing values. Quantitative variables were described using mean and standard deviation or median and interquartile range (IQR) values when normality conditions were not met. Categorical variables were presented as absolute numbers and percentages.

The univariate statistical analysis of the quantitative variables, with independent groups, was performed using the Student *t* test whenever its application conditions were met; otherwise, the Mann–Whitney or Kruskal–Wallis *U* test was applied. For categorical variables, the Pearson χ^2^ test or Fisher’s exact test was used, depending on the conditions of application. A value of *p* < 0.05 and a confidence interval of 95% were considered statistically significant whenever the conditions were met.

A multivariate logistic regression analysis was performed with anastomotic stenosis as dependent variable and introducing the statistically significant variables or those with a trend towards significance with *p* < 0.2.

## Results

During the study period (2012–2022), after applying the selection criteria, a total of 239 patients were included: 172 from the Hospital Universitari Parc Taulí and 67 from the Consorci Sanitari de Terrassa.

Table 1 shows the variables of the patients who suffered BAS, which was detected in 39 patients (16.3%). The majority of patients in the study were men (170, 71.1%), 29 were smokers (12.2%), 55 diabetics (23.2%) and 12 suffered vascular disease (5.1%). The median age was 67 years and the predominant ASA score was I/II (61%). The median distance from the tumor to the anal verge was 9 cm (interquartile range 2) and the median tumor size was 4.5 cm. Most lesions (87, 36.7%) occupied two quadrants and 148 (62.2%) patients received neoadjuvant treatment. Regarding the surgical variables, laparoscopic surgery, LAR technique, end-to-end anastomosis and CMS diameter 28–29 mm were the most frequently used. Regarding the postoperative variables, 160 (66.9%) patients presented morbidity, but in only 26 (10.9%) was it relevant (Clavien-Dindo grade > II).

**Table 1:** Stenosis variables

		All patients *n* = 239	Stenosis *n* = 39 (16.3%)	No stenosis *n* = 200 (83.7%)	*P* value Difference (95% CI)
Demographic and patient variables					
Sex (*n* = 239)	Women	69 (28.9)	11 (28.2)	58 (29)	1 (− 10.81 to 9.75)
	Men	170 (71.1)	28 (71.8)	142 (71)	
Age, median (IQR) years		67 (15)	66 (18)	67.5 (14)	0.537
Smoker (*n* = 237)	Yes	29 (12.2)	2 (5.1)	27 (13.6)	0.184 (− 21.48 to − 0.31)
Diabetes (*n* = 237)	Yes	55 (23.2)	8 (20.5)	47 (23.7)	0.836 (− 13.29 to 8.31)
Vasculopathy (*n* = 237)	Yes	12 (5.1)	3 (7.7)	9 (4.5)	0.423 (− 15.96 to 33.96)
BMI, median (IQR)		27 (6.1)	26.4 (7.8)	27 (5.9)	0.441*
Albumin (g/L), median (IQR)		43 (5)	42.9 (5.1)	43.2 (4.9)	0.854*
ASA score (*n* = 239)	I/II	146 (61)	24 (61.5)	122 (61)	1 (− 9.28 to 9.9)
	III/IV	93 (39)	15 (38.5)	78 (39)	
Immunosuppression (*n* = 237)	Yes	3 (1.3)	0 (0)	3 (1.5)	1 (− 20.96 to − 11.51)
Distance from anal verge (cm), median (IQR)		9 (2)	9 (3)	9 (2)	0.872*
Tumor size (cm), median (IQR)		4.5 (2)	5 (2.5)	4.5 (2)	0.314*
Quadrants (*n* = 237)	1	33 (13.9)	5 (12.8)	28 (14.1)	0.802*
	2	87 (36.7)	16 (41)	71 (35.1)	
	3	36 (15.2)	7 (19.4)	29 (14.6)	
	4	81 (34.2)	11 (28.2)	70 (35.4)	
Neoadjuvant treatment (*n* = 238)	Yes	148 (62.2)	19 (48.7)	129 (64.8)	0.071 (− 19.52 to 0.76)
Surgical variables					
Approach (*n* = 239)	Open/conversion	3 (1.3)	2 (5.1)	1 (0.5)	0.746 (− 9.04 to 20.24)
	Laparoscopy	217 (90.8)	35 (89.7)	182 (91)	
	Robot	19 (7.9)	2 (5.1)	17 (8.5)	
Surgical technique (*n* = 239)	TaTME	82 (34.3)	15 (38.5)	67 (33.5)	0.583 (− 7.08 to 13.09)
	LAR	157 (65.7)	24 (61.5)	133 (66.5)	
Anastomosis diameter (*n* = 239)	28–29 mm	165 (69)	35 (89.7)	130 (66)	0.002 (7.27 to 23.53)
	31–33 mm	74 (31)	4 (10.3)	70 (34)	
Anastomosis type (*n* = 234)	TT	199 (85)	30 (78.9)	169 (84.9)	0.318 (− 22.56 to 6.99)
	TL	35 (15)	8 (21.1)	27 (77.1)	
Surgery time (min), median (IQR)		240 (90)	255 (120)	240 (95)	0.962*
Leak (*n* = 238)	Yes	46 (19.3)	7 (17.2)	39 (19.6)	1 (− 13.09 to 10.19)
Postoperative variables (30 days)					
Readmission (*n* = 239)		32 (16.3)	7 (17.9)	25 (12.5)	0.439 (− 8.73 to 21.56)
Reintervention (*n* = 239)		7 (2.9)	0 (0)	7 (3.5)	0.603 (− 21.62 to − 12)
Dehiscence (*n* = 237)		22 (9.3)	4 (10.3)	18 (9.1)	0.767 (− 14.95 to 18.76)
Overall morbidity (*n* = 239)		160 (66.9)	30 (76.9)	130 (65)	0.193 (− 26.71 to 2.86)
Clavien (relevant > II) (*n* = 239)		26 (10.9)	4 (10.3)	22(11)	1 (− 9.72 to 11.21)
CCI, median (IQR)		21 (17)	19 (17)	21 (17)	0.9*
Time until ileostomy closure (months), median (IQR)		2.5 (8)	7 (13)	2 (7)	0.02
Ileostomy closure (*n* = 236)		181 (76.7)	30 (78.9)	151 (76.3)	0.836 (− 8.75 to 12.81)
Clavien-Dindo morbidity (*n* = 163)	I	45 (28.1)	34 (26.2)	11 (36.7)	0.806
	II	89 (55.6)	74 (56.9)	15 (50)	
	IIIa	10 (6.3)	8 (6.2)	2 (6.7)	
	IIIb	9 (5.6)	8 (6.2)	1 (3.3)	
	IVa	7 (4.4)	6 (4.6)	1 (3.3)	
	IVb	0 (0)	0 (0)	0 (0)	
	V	3 (1.2)	0 (0)	3 (1.5)	

*IQR* Interquartile range, *BMI* body mass index, *CI* confidence interval

*Mann–Whitney *U* test

Assessing groups according to the presence or absence of stenosis (Table 1), no significant differences were found in patient and demographic variables. Regarding the surgical variables, only CMS diameter (28–29 vs 31–33 mm) presented significant differences (*p* = 0.002, 95% CI 7.27–23.53). Nor were significant differences found in the mid-term postoperative variables, except for time of ileostomy closure, which was longer in the stenosis group (*p* = 0.02). Table 2 compares the 165 patients in whom the shorter CMS was used vs. the 74 patients in whom the longer CMS was used. With regard to the demographic and patient variables, the 31–33 mm suture was used significantly more often in men, in smokers and in patients undergoing neoadjuvant therapy, and with regard to the surgical variables, it was used significantly more frequently when TaTME or end-to-end anastomosis were performed. As for immediate postoperative complications, readmissions were higher in the 31–33 mm CMS group, but no differences were found regarding morbidity or anastomotic leaks. In the medium-term follow-up, BAS was detected significantly more often in the 28–29 mm group (35/165 patients, 21%) compared with four out of 74 (5.6%) in the 31–33 mm group (*p* = 0.002, 95% CI 7.27–23.53). No differences were found between the groups regarding the Truong BAS types. In the 28–29 mm CMS group, ileostomy closure took longer (median 6 months, IQR 10, *p* =  < 0.01) and was achieved less often (121/165, 72.5%) than in the 31–33 mm group (62/74 (86.1%), *p* = 0.03 (95% CI − 24.13 to − 3.18).

**Table 2:** Diameter size anastomosis variables

		All patients *n* = 239	28–29 mm diameter *n* = 165 (69%)	31–33 mm diameter *n* = 74 (31%)	*P* value Difference (95% CI)
Demographic and patient variables					
Sex (*n* = 239)	Women	69 (28.9)	55 (32.7)	14 (18.9)	0.031 (− 26.24 to − 2.55)
	Men	170 (71.1)	111 (65.3)	59 (81.1)	
Age, median (IQR) years		67 (15)	68 (15)	65 (13)	0.164*
Smoker (*n* = 237)	Yes	29 (12.2)	13 (7.8)	16 (21.9)	0.004 (9.09 to 47.22)
Diabetes (*n* = 237)	Yes	55 (23.2)	41 (24.9)	14 (19.2)	0.324 (− 20.37 to 5.76)
Vasculopathy (*n* = 237)	Yes	12 (5.1)	11 (6.6)	1 (1.4)	0.113 (− 40.01 to − 6.48)
BMI, median (IQR)		27 (6.1)	26.8 (6.33)	27.6 (5.49)	0.584 (− 2 to 0.51)
Albumin (g/L), median (IQR)		43 (5)	42.9 (5)	44 (4.8)	0.04*
ASA score (*n* = 239)	I/II	146 (61)	98 (58.2)	48 (61.5)	0.318 (− 5.2 to 18.23)
	III/IV	93 (39)	69 (40.1)	25 (33.8)	
Immunosuppression (*n* = 237)	Yes	3 (1.3)	1 (0.6)	2 (2.8)	0.215 (− 16.53 to 90.79)
Distance from anal verge (cm), median (IQR)		9 (2)	9 (2)	8 (2)	0.009*
Tumor size (cm), median (IQR)		4.5 (2)	4.5 (1.7)	5 (2.5)	0.174*
Quadrants (*n* = 237)	1	33 (13.9)	26 (15.6)	7 (9.6)	0.571
	2	87 (36.7)	57 (34.3)	30 (41.1)	
	3	36 (15.2)	24 (14.7)	12 (16.4)	
	4	81 (34.2)	57 (34.3)	24 (32.9)	
Neoadjuvant treatment (*n* = 238)	Yes	148 (62.2)	94 (56.2)	54 (74)	0.014 (4.28 to 26.9)
Surgical variables					
Approach (*n* = 239)	Open/conversion	3 (1.3)	3 (1.8)	0 (0)	0.067
	Laparoscopy	217 (90.8)	146 (88)	71 (97.3)	
	Robot	19 (7.9)	17 (10.2)	2 (2.7)	
Surgical technique (*n* = 239)	TaTME	82 (34.3)	69 (41.6)	13 (17.8)	< 0.001 (− 33.58 to − 11.83)
	LAR	157 (65.7)	97 (58.4)	60 (82.2)	
Anastomosis type (*n* = 234)	TT	199 (85)	131 (78.9)	68 (93.2)	0.018 (6.56 to 33.18)
	TL	35 (15)	30 (18.1)	5 (6.8)	
Surgery time (min), median (IQR)	240 (90)	255 (120)	215 (101)	0.06	
Leak (*n* = 238)	Yes	46 (19.3)	39 (23.2)	7 (9,6)	0.13 (− 30.95 to − 6.31)
Postoperative variables (30 days)					
Readmission (*n* = 239)	Yes	32 (13.4)	18 (10.9)	14 (19.4)	0.024 (2.63 to 38.83)
Reintervention (*n* = 239)	Yes	7 (2.9)	5 (3)	2 (2.7)	1 (− 36.05 to 31.91)
Dehiscence (*n* = 237)	Yes	22 (9.3)	16 (10.2)	6 (8.5)	0.813 (− 23.11 to 14.82)
Overall morbidity (*n* = 239)	Yes	160 (66.9)	118 (69.6)	45 (59.4)	0.185 (− 4.29 to 21.95)
Clavien (relevant > II) (*n* = 239)	Yes	26 (10.9)	18 (10.7)	8 (10.8)	1 (− 8.43 to 8.62)
CCI, median (IQR)		21 (17)	1 (24)	21 (27)	0.06*
Morbidity. Clavien-Dindo (*n* = 163)	I	45 (28.1)	34 (29.1)	11 (24.4)	0.136
	II	89 (55.6)	64 (55.6)	25 (57.8)	
	IIIa	10 (6.3)	5 (4.3)	5 (11.1)	
	IIIb	9 (5.6)	9 (7.7)	0 (0)	
	IVa	7 (4.4)	4 (3.4)	3 (6.7)	
	IVb	0 (0)	0 (0)	0 (0)	
	V	3 (1.2)	2 (1.2)	1 (1.4)	
Follow up mid-term					
Time until ileostomy closure (months), median (IQR)		2.5 (8)	6 (10)	1 (1)	< 0.01*
Ileostomy closure (*n* = 239)		183 (76.6)	121 (72.5)	62 (86.1)	0.03 (− 24.13 to − 3.18)
Stenosis anastomosis		39 (16.3)	35 (21)	4 (5.6)	0.002 (7.27 to 23.53)
Stenosis type *n* = 39 (16.3%)	I *n* = 25 (64%)		22 (62.9)	3 (75)	0.605
	II *n* = 7 (18%)		6 (17.1)	1 (25)	
	III *n* = 7 (18%)		7 (20)	0 (0)	

*IQR* Interquartile range, *BMI* body mass index, *CI* confidence interval

*Mann–Whitney *U* test

The multivariate binary logistic regression analysis aimed to identify predictors of BAS, including all variables that obtained a *p* < 0.2 in the univariate analysis. The only predictor found was CMS diameter, with an odds ratio (OR) of 3.5 (95% CI 1.2–10.5) in favor of the larger diameter stapler.

## Discussion

BAS is a frequent complication of colorectal anastomosis after surgery for neoplastic or inflammatory disease, with an incidence varying between 2 and 30%. The use of different criteria for its definition may be the reason for this wide variation [5, 6, 23, 25]. BAS is associated with mucosal/submucosal lesions due to tissue hemorrhage or edema, which, by activating an inflammatory process, may cause long-term tissue fibrosis. BAS is not a negligible complication; it can cause fecal urgency or incontinence, and in extreme cases intestinal obstruction, and may significantly impact the quality of life of patients even years after surgery [26]. Endoscopic dilation is considered the treatment of choice for BAS and although recurrence is frequent, repeated sessions are generally effective. When surgical treatments are required, these consist of radial diathermy incisions, repeat anastomosis or, less frequently, the creation of a stoma.

BAS is the second most serious complication associated with anastomoses after rectal cancer resection [27]. However, studies analysing the frequency of occurrence and its related factors are scarce. Our study was based on a sample with sufficient power to assess the variability described in the literature [5, 6, 23, 25]. In accordance with previous studies, the frequency of 15% was chosen for its calculation [23], requiring a minimum of 210 patients; our final sample comprised 239 subjects. BAS was detected in 39 patients (16.3%), a rate similar to the one recorded in the meta-analysis by He et al. [25], who reported a rate of 17% in 659 patients. Truong et al. [19]’s classic definition of BAS was used, as it combines objective measurements of the size of the stenosis and its symptoms. Regarding the risk factors for BAS, He et al. [25] reported associations with younger age, male sex, radiotherapy, protective ileostomy, intersphincteric anastomosis, suture failure and distance of the anastomosis from the anal verge; in contrast, those authors observed that CMS had a protective effect. We sought to identify the risk factors for BAS through a logistic regression analysis that included the factors with *p* < 0.2 in the univariate analysis, namely smoking, neoadjuvant treatment and stapler diameter; of these, only CMS emerged as a predictive risk factor, with an OR of 3.5 and 95% CI of 1.2–10.5. Anastomotic leak was not related to the appearance of BAS. Anastomoses using CMS were associated with less tissue trauma, and as has been described elsewhere, staple use had a protective effect against BAS [25]. However, the diameter of the CMS varies widely, the most commonly used in LAR being 28–31 mm. The choice of the CMS diameter depends on the diameter of the left colon, as it is important not to tear the tissue when introducing the anvil. But this choice is often intuitive, and is difficult to make even for an experienced surgeon. An incorrect choice of stapler lengthens surgical time and increases the manipulation of the colon, leading in some cases to ischemia or perforation and increasing the cost of the intervention, since the stapler must be replaced with a smaller one and cannot be reused [28–31].

Given the difficulty of deciding on the diameter of the CMS to be used and the importance of adequate selection due to its impact on postoperative complications, this study compared two groups of patients with CMS of 28–29 vs 31–33 mm. It was found that the larger diameter suture was used in males, smokers, in patients requiring neoadjuvant treatment and when an end-to-end anastomosis was performed. As mentioned above, in our series different demographic and surgical variables were analyzed in relation to postoperative surgical stenosis. A statistically significant relationship was only obtained with the diameter of the anastomosis and the rate and time until ileostomy closure. The 28–29 mm stapler was associated with the appearance of BAS, longer time for the closure of the ileostomy and a lower rate of ileostomy reconstruction. In contrast, neither CMS size was related to a higher previous morbidity or anastomotic dehiscence in the immediate postoperative period.

Since there was no significant relationship with neoadjuvant treatment in these patients, the relationship with each type of neoadjuvant treatment was not analyzed.

Few studies have evaluated the BAS according to the diameter of the stapler used. In a study on anastomoses in the left colon and rectum, Reif de Paula et al. concluded that the use of 25–29 mm CMS was associated with an increase in BAS compared to 30–33 mm CMS (7.1 vs 2.1%, *p* = 0.007). They did not show significant differences in relation to dehiscence or reintervention [12]. In a study of the relationship of the diameter of the CMSs with anastomotic dehiscence in LAR, Jiang et al. [2] did not observe a relationship with BAS, although this was not the objective of their analysis. In other areas of surgery, such as esophagectomy with cervical anastomosis, the presence of cervical BAS has also been related to a smaller CMS diameter [32].

Staplers measuring 31–33 mm cannot always be used in an LAR in the left colon. Various devices have been described for the dilation of the proximal colonic end of an anastomosis, for example Fazio Colorectal Cleveland Clinic Intestinal Dilators [33], circular stapler anvil sizer set (TFS-Tools For Surgery) [34], and Ethicon Circular sizers [35]. All these devices are designed for open surgery and are based on mechanical dilation of the colon wall to facilitate the introduction of the CMS.

Our research institute (I3PT) has created a device designed to solve the problem of dilation, which is currently in the preclinical validation phase. The device, which can be used in both laparoscopic/robotic and open surgery, achieves dilation not only through mechanical methods but through other means that do not affect the colon wall, thus enabling the use of a stapler with the correct diameter.

The main limitation of the present study is its retrospective design, despite the prospective introduction of data. The absence of randomization in the CMS groups may introduce a bias, since the diameter of the anastomosis was chosen by the surgeon in each patient on an individual basis, a decision influenced by the surgeon’s own personal experience (and therefore subjective). In addition, the data come from two different hospitals with numerical disparity (172 vs 67 patients) which may introduce another bias. The main strength of the study was the analysis of a topic that has attracted very little attention in the literature, namely BAS in relation to the CMS diameter in an adequately powered sample.

## Conclusion

The rate of BAS after LAR was not negligible, affecting 39 out of 239 patients (16.3%). The use of a CMS with a diameter of 31–33 mm was associated with a lower rate of BAS, which was recorded in four out of 74 patients (5.6%), and with fewer overall postoperative complications, fewer ileostomies and a shorter time to closure, compared with CMS of 28–29 mm. In contrast, no association was established between the diameter of the CMS and anastomotic dehiscence.

Our results appear to favor the use of larger diameter CMS and dilation devices to prevent BAS. However, clinical trials are required to confirm these findings.
